# Cytogenomic Evaluation of Subjects with Syndromic and Nonsyndromic Conotruncal Heart Defects

**DOI:** 10.1155/2015/401941

**Published:** 2015-06-07

**Authors:** Karen Regina de Souza, Rafaella Mergener, Janaina Huber, Lucia Campos Pellanda, Mariluce Riegel

**Affiliations:** ^1^Postgraduate Program in Genetics and Molecular Biology, Universidade Federal do Rio Grande do Sul (UFRGS), 91501-970 Porto Alegre, RS, Brazil; ^2^Institute of Cardiology, University Foundation of Cardiology, Avenida Princesa Isabel 297, 90620-000 Porto Alegre, RS, Brazil; ^3^Medical Genetics Service, Hospital de Clínicas de Porto Alegre, Rua Ramiro Barcelos 2350, 90035-903 Porto Alegre, RS, Brazil

## Abstract

Despite considerable advances in the detection of genomic abnormalities in congenital heart disease (CHD), the etiology of CHD remains largely unknown. CHD is the most common birth defect and is a major cause of infant morbidity and mortality, and conotruncal defects constitute 20% of all CHD cases. We used array comparative genomic hybridization (array-CGH) to retrospectively study 60 subjects with conotruncal defects and identify genomic imbalances. The DNA copy number variations (CNVs) detected were matched with data from genomic databases, and their clinical significance was evaluated. We found that 38.3% (23/60) of CHD cases possessed genomic imbalances. In 8.3% (5/60) of these cases, the imbalances were causal or potentially causal CNVs; in 8.3% (5/60), unclassified CNVs were identified; and in 21.6% (13/60), common variants were detected. Although the interpretation of the results must be refined and there is not yet a consensus regarding the types of CHD cases in which array-CGH should be used as a first-line test, the identification of these CNVs can assist in the evaluation and management of CHD. The results of such studies emphasize the growing importance of the use of genome-wide assays in subjects with CHD to increase the number of genomic data sets associated with this condition.

## 1. Introduction

Congenital heart defects are defined as structural abnormalities of the heart or intrathoracic vessels and represent one of the most common congenital anomalies at birth. Congenital heart disease (CHD) affects approximately 81.4 of every 10,000 live births (LBs) [1] and is responsible for a significant proportion of cases of infant morbidity and mortality [2–4]. Conotruncal heart malformations comprise approximately 20% of all CHD cases [5, 6]. The most common conotruncal heart defects are cardiac outflow tract defects, such as Tetralogy of Fallot (TOF), transposition of the great arteries (TGA), double outlet right ventricle, truncus arteriosus communis, and aortic arch anomalies. TOF [OMIM number 187500] is the most common cyanotic CHD phenotype and occurs in 1 in 2,500 LBs [7]. In the past, 80% of children born with TOF died before their tenth birthday; however, as medicine has advanced and early detection has permitted cardiac surgery, many of these newborns survive and contribute to an increased population of adults with CHD [8].

Environmental and genetic factors, including recognized chromosomal and Mendelian syndromes, have been cited as causes of CHD [9–16]. However, nonsyndromic, non-Mendelian factors contribute to the largest proportion of CHD cases [17]. In the past decade, several genomic studies on isolated and syndromic CHD cases have shown that mutations in candidate genes and chromosomal regions can predispose patients to the development of several types of CHD [18, http://homes.esat.kuleuven.be/~bioiuser/chdwiki/index.php/Main_Page]. The most frequent appears to be the 1q21.1 duplication, which varies somewhat in size and coverage. This CNV occurs in at least 1% of reported CHD cases. The largest study concluded that the 1q21.1 duplication was associated specifically with TOF and that the GJA5 gene was involved in all CHD cases [19]. Three types of CNVs can be distinguished among those found in CHD: (1) CNVs associated with well described microdeletion syndromes that include CHD (e.g., 22q11.2 deletion syndrome and William's syndromes) or partial aneuploidy due to chromosomal rearrangements; (2) CNVs that include genes known or likely to be involved in heart development, for example, GATA4(8p23.1-p22) and NODAL(10q22.1); and (3) CNVs associated with a wide variety of other phenotypes such as autism or schizophrenia, which often show reduced penetrance or inheritance from an unaffected parent [18, http://homes.esat.kuleuven.be/~bioiuser/chdwiki/index.php/Main_Page].

DNA microarray-based technology permits the detection of submicroscopic copy number variants (CNVs) in the genome and has been widely used in clinical practice. Array comparative genomic hybridization (array-CGH) was initially used to distinguish candidate genes involved in the pathogenesis of cancer and to identify chromosomal imbalances by detecting CNVs in tumors [20, 21]. In clinical diagnostics, both oligonucleotide array-CGH and single-nucleotide polymorphism (SNP) genotyping have been shown to be powerful genomic methods for evaluating idiopathic mental retardation (MR) [also referred to as developmental delay (DD), intellectual disability (ID), or learning difficulty] and its associated congenital abnormalities (MCA), autistic spectrum disorders (ASDs), schizophrenia, and other neuropsychiatric disorders [22]. Furthermore, comparative genomic analysis using array-CGH has been increasingly used to detect genomic imbalances involving loci and genes with known roles in cardiac development, and this approach may also identify candidate genes related to CHD [23–26]. Indeed, array-CGH can detect pathogenic genomic imbalances, and this application may be especially valuable for subjects with CHD of unknown etiology.

Therefore, the aim of this retrospective study was to use whole-genome microarray-based CGH analysis to identify genomic imbalances that are likely to be associated with conotruncal heart defects. Between January 2013 and May 2014, we selected and analyzed 60 stored DNA samples from patients with conotruncal heart defects of unknown cause. In addition, based on evaluations by a cardiology reference center in the state of Rio Grande do Sul, Brazil, this study evaluated the usefulness of array-CGH as a diagnostic tool for patients with CHD.

## 2. Materials and Methods

### 2.1. Sample Selection

This retrospective study was performed using DNA extracted from blood samples of patients whose identifying information had been removed, and the samples were obtained from the biorepository of the Institute of Cardiology, University Foundation of Cardiology, Brazil. The Institute of Cardiology is a regional referral center in the state of Rio Grande do Sul, southern Brazil. Venipuncture was used to collect 5 mL of blood from each patient. The Lahiri and Nurnberg method was used for DNA extraction. In cases for which the collection of a new blood sample was necessary, DNA extraction was performed with a Pure Link Genomic DNA kit (Invitrogen, São Paulo, Brazil). The subjects were between 22 days and 33 years of age at the time of blood collection, and they presented congenital cardiac anomalies of unknown cause. At the time of the comparative genomic analysis, the patients' clinical and laboratory data from their first referrals were collected from the hospital's records. The data did not include follow-up investigations or disease outcomes. Cases without sufficient clinical data were excluded, as were cases in which the mothers had suspected infectious or parasitic diseases or a history of illicit drug or alcohol use during pregnancy. Individuals with double outlet right ventricle or atrioventricular septal defects that accompanied TOF or TGA were excluded. We also excluded DNA samples from subjects with a known genetic disorder, major congenital anomalies, and/or chromosomal imbalances that had been detected in previous studies [27]. To avoid contamination of the donor DNA, the study also excluded subjects who had received blood transfusions in the 4 months prior to blood collection [28]. Based on these criteria, a total of 68 samples were selected, but eight of the samples were excluded because the quality of the extracted DNA was insufficient for array-CGH analysis. Therefore, the study was carried out with 60 samples. All of the patients or their guardians provided informed consent before their inclusion in the study. The study protocol was approved by the Institutional Ethics Committee and was conducted in accordance with current institutional ethics rules regarding the use of biological materials from biorepositories [29].

### 2.2. Whole-Genome Array-CGH

We performed comparative genomic analysis with oligonucleotide array-based CGH using an 8 × 60 K whole-genome platform (design 021924, Agilent Technologies, Santa Clara, California, United States of America), with an average distance of 40 kb between probes. Genomic DNA was isolated from the peripheral blood of 60 subjects (available at the biorepository) and subsequently analyzed. For each experiment, a gender-mismatched normal reference (Promega Corp., Madison, WI, USA) was used. The experiments were performed according to the manufacturer's protocol. Images of the arrays were taken using a microarray scanner (G2600D) and processed using the Feature Extraction software (v 9.5.1), both from Agilent. As to the measurement of data quality, various quality control (QC) parameters have been devised and included both in software packages commercial and in the public domain. These QC parameters calculate microarray data noise, appreciation of which is critical to matters like false discovery rate. For those CGH arrays manufactured by* Agilent Technologies*, as used in our study, the major QC parameter is known as the derivative log ration, or DLR. In our study DRL values of <0.15 were indicative of acceptable data. The raw data were analyzed using the Agilent Cytogenomics v2.7.8.0 software and the ADM-2 statistical algorithm (second generation algorithm that can assist in controlling noise source), with a threshold of 6.0 and a 4-probe minimum aberration call. Subsequent normalization of the data was performed using the software to verify changes in DNA copy number. The *P* values for each probe were calculated, providing additional objective statistical criteria to determine if each probe's deviation from zero was statistically significant [30]. All experiments included two array hybridizations per sample, and the results were recorded and compared. To exclude false positive results, we confirmed the detected deletions and duplications using dye-swap experiments. Laboratory personnel were blinded to prior testing results. Array CGH detected all known regions of genomic imbalance in 10 validation samples with 100% concordance and an excellent signal-to-noise ratio (<0.1). Only genomic imbalances that were detected in both dye-swap experiments were reported.

### 2.3. Data Analysis

Whole-genome array-CGH data analysis was performed in a blinded fashion. Specifically, the samples were obtained, the identifying information was removed, and the investigators who performed the array-CGH analyses were not aware of the prior clinical and/or laboratory information related to each sample. The DNA CNVs that were detected were compared with the CNVs that had been reported in at least 3 publicly available online resources and in databases of chromosomal abnormalities and variants. Our own in-house database was also consulted but was not regarded as conclusive,* per se*. We classified the CNVs (gains/duplications and losses/deletions) into the following categories: common or benign CNVs (normal genomic variants); CNVs of uncertain clinical relevance or unclassified (variants of uncertain significance (VOUS)); and pathogenic CNVs of clinical relevance (causal or potentially causal variants). Pathogenic refers to CNV reported in the medical literature, or publicly available databases, as being associated with known disease and likely to be clinically significant; VOUS refers to CNV that has not reported in the medical literature or listed in publicly available databases as being associated with known disease and benign refers to CNV recorded and/or curated in publicly, or in-house, genomic databases as polymorphic variants without known effect among control individuals. In this study, the causal or pathogenic CNVs included those in known pathogenic regions, deletions, and duplications of >3 Mb or that were visible by G-banded karyotyping and had not been reported in the normal population, and microdeletions or microduplications of <3 Mb that had previously been reported as causal. Common deletions or duplications included variants that were well documented in the normal population or were previously reported as polymorphisms. Deletions or duplications were classified as being VOUS or unclassified when insufficient evidence was available to conclude if the CNV was either a causal or a common variant. As a reference, we used public data from compiled, collaborative databases including the Clinical Genomic Resource (ClinGen) (http://clinicalgenome.org/); the Congenital Heart Defects (CHD) Wiki (http://homes.esat.kuleuven.be/~bioiuser/chdwiki/index.php/Main_Page); the Database of Chromosomal Imbalance and Phenotype in Humans Using Ensembl Resources (Decipher) (http://decipher.sanger.ac.uk/); the European Cytogeneticists Association Register of Unbalanced Chromosome Aberrations (ECARUCA) (http://umcecaruca01.extern.umcn.nl:8080/ecaruca/ecaruca.jsp); the Ensembl Genome Browser (http://www.ensembl.org/index.html); the National Center for Biotechnology Information (NCBI) (http://www.ncbi.nlm.nih.gov/); and the University California Santa Cruz (UCSC) Genome Browser (http://genome.ucsc.edu/).

## 3. Results and Discussion

Data on the 60 subjects included in this study, who all had conotruncal heart defects of unknown cause and whose DNA, was analyzed via whole-genome array-CGH, are presented in Table 1. We determined that 36 of the 60 subjects were born with TOF, 22 were born with TGA, and 2 were born with truncus arteriosus. Of the 60 subjects, 35 (58.3%) were male and 25 (41.7%) were female. The ages of the subjects ranged from 22 days to 33 years at the time of blood collection (mean 8.2 years; standard deviation 4.0 years), and 51 (85%) of them were infants. We identified 23/60 (38.3%) cases with DNA CNVs. Overall, microdeletions were verified in 14/60 (23.3%) cases, and microduplications were verified in 9/60 (15%) cases. A total of 10/60 (16.6%) subjects were identified as having significant genomic imbalances; we identified clinically significant chromosomal imbalances or causal CNVs in 5/10 (50%) of these cases. Phenotypically, 8/10 (80%) subjects were identified as having TOF, and 2/10 (20%) individuals had TGA. The details of the array-CGH results from the cases with relevant genomic imbalances (causal and/or variant of uncertain significance) are summarized in Table 2.

**Figure 1:**
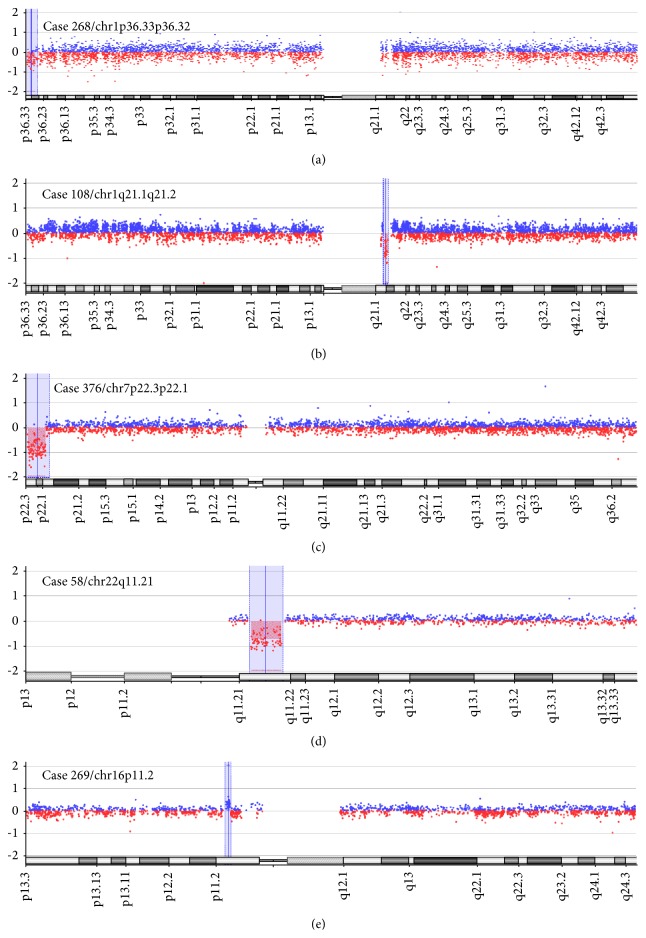
Array-CGH ratio profiles of chromosomes from 6 subjects with pathogenic or potentially pathogenic genomic imbalances using genomic DNA from the patients as a test (red) and DNA from normal subjects as a reference (blue). The test/reference ratio data for each chromosome is shown. Each dot represents a single probe (oligo) spotted on the array. The log ratio of the chromosome probes is plotted as a function of chromosomal position. A copy number loss shifts the ratio downward (approximately −1x); a copy number gain shifts the ratio upward (approximately +1x). The ideogram of each chromosome (bellow each profile) shows the location of each probe. The probe log2 ratios were plotted according to genomic coordinates (based on the UCSC Genome Browser, February 2009, NCBI Build 37 reference sequence). (a) An approximately 2.94 Mb terminal deletion at chromosome 1p36.33-p36.32 (blue box) in case 268. (b) An approximately 1.15 Mb interstitial deletion at chromosome 1q21.1-q21.2 (blue box) in case 108. (c) An approximately 4.56 Mb terminal deletion at chromosome 7p22.3p22.1 (blue box) in case 376. (d) An approximately 2.5 Mb interstitial deletion at chromosome 22q11.21 (blue box) in case 58. (e) An approximately 0.52 Mb interstitial duplication at chromosome 16p11.2 (blue box) in case 269.

**Figure 2:**
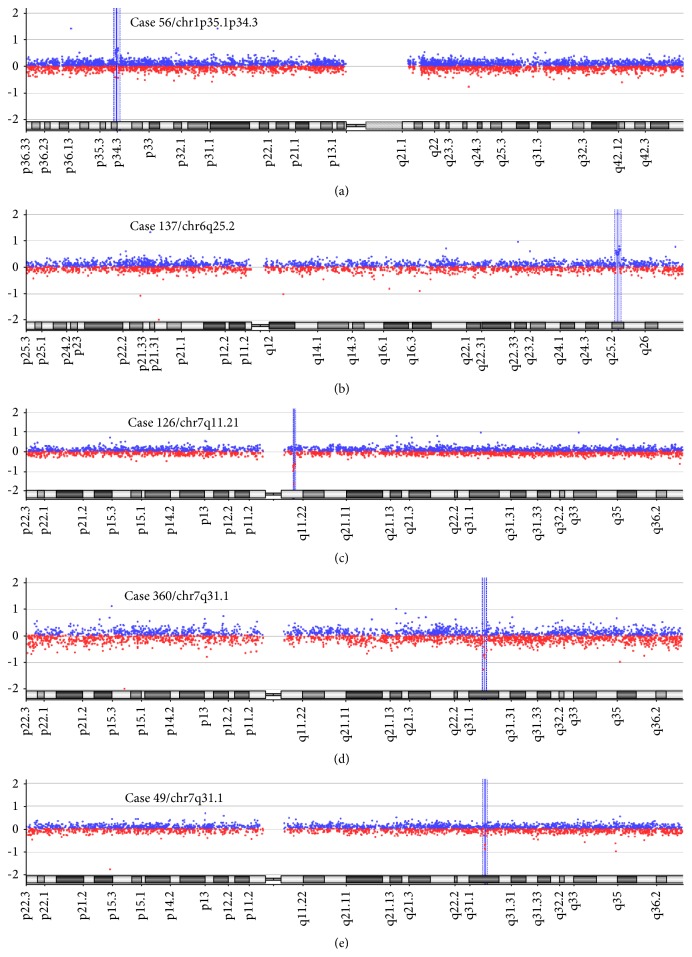
The array-CGH profiles of a series of variants of uncertain significance in several cases. (a) An approximately 0.88 Mb duplication at chromosome 1p35.1p34.3 (blue box) in case 56. (b) An approximately 1.0 Mb duplication at chromosome 6q25.2 (blue box) in case 137. (c) An approximately 0.37 Mb deletion at chromosome 7q11.21 (blue box) in case 126. (d) An approximately 0.22 Mb deletion at chromosome 7q31.1 (blue box) in case 360. (e, d) An approximately 0.10 Mb deletion at chromosome 7q31.1 (blue box) in case 49.

Using array-CGH, we identified seven microdeletions (1p36.33-p36.32; 1q21.1-q21.2; 7p22.3-p22.1; 7q11.22; 22q11.2; and 2 cases with 7q31.1) and three microduplications (1p35.1-p34; 6q25.2; and 16p11.2). Of these ten potentially meaningful CNVs, the deletions were classified as causal or potentially causal in 4 cases (268, 108, 376, and 58) and as VOUS in 3 cases (126, 360, and 49). The duplications were classified as causal in 1 case (269) and as VOUS in 2 cases (56 and 137). Variants that are not recurrently found in normal individuals were considered causal when they contained dosage-sensitive genes whose loss-of-function mutations are known to cause syndromic or nonsyndromic conotruncal heart defects or genes that cause recessive forms of CHD. Examples of graphical overviews of the array-CGH data are shown in Figures 1 and 2.

Among the 8 subjects with TOF who also had detectable genomic imbalances, 2 cases (268 and 58) exhibited clinically significant microdeletions that coincided with the well-described 1p36 deletion syndrome [OMIM:607872] and with 22q11.2 deletion syndrome [OMIM  number  188400/number  192430, 2007], respectively. In these two cases with CNVs associated with well-defined genetic disorders, the genomic imbalances could have been previously diagnosed by, for example, fluorescence* in situ* hybridization (FISH) analysis (using locus-specific probes for the critical chromosome region) or multiplex ligation-dependent probe amplification (MLPA) analysis using markers within the critical segments, if the clinical findings at the time of referral were indicative of a particular microdeletion syndrome that could inform exactly which region(s) and/or chromosome(s) to investigate. However, both samples were from subjects in whom neither FISH nor MLPA analysis was previously performed. Because there are several genomic imbalances that are associated with types of syndromic CHD whose causal genes have not yet been identified, it is necessary to first look for known chromosomal syndromes that have previously been described. The phenotypic characteristics of well-defined microdeletion/microduplication syndromes associated with conotruncal heart defects can often be clinically detected before the causal microdeletions are identified [31]. However, the clinical evaluation of individuals with such syndromes continues to challenge clinicians and requires a high degree of experience and expertise. Although some diagnostic steps are highly standardized (e.g., database searches, clinical utility gene cards, and standard clinical scores), others are not suitable for standardization. Moreover, the diagnosis of microdeletion/microduplication syndromes using only clinical assessment may be difficult because of the great variability in the symptoms, especially relative to the size of the genomic imbalance and the expertise of the clinician [32]. In contrast to single gene disorders, contiguous gene deletions (which are often associated with CHD), and especially those resulting in developmental delays, intellectual disabilities, or congenital developmental abnormalities, are caused by submicroscopic chromosomal rearrangements that encompass several genes; generally, at least two of these genes are dosage-sensitive but functionally unrelated [33]. There are also syndromes that do not become distinct until a certain age, at which time a particular behavior or clinical manifestation presents. Because of the growing number of recognized genetic syndromes and chromosome abnormalities and because of the overlapping clinical characteristics of these syndromes, it is becoming increasingly difficult to use only a clinical examination to determine exactly which syndrome affects an individual with syndromic CHD. In syndromes with CHD as part of the clinical spectrum, conotruncal heart defects are often the first symptoms to appear [34].

An additional 3 subjects with TOF showed imbalances involving a 1.15 Mb deletion in the chromosome 1q21.1-q21.2 region (case 128), a 4.56 Mb deletion in the 7p22.3-p22.1 region that could be associated with a chromosomal syndrome (case 376), and a 0.52 Mb duplication in the chromosome 16p11.2 region (case 269). Agergaard et al. [34] examined the 1q21.1 locus in 948 patients with TOF, 1,488 patients with other forms of CHD, and 6,760 ethnically matched controls using SNP genotyping arrays (Illumina 660W and Affymetrix 6.0) and multiplex ligation-dependent probe amplification. Recurrent rearrangements of chromosome 1q21.1 have been associated with variable phenotypes that exhibit incomplete penetrance, including CHD. The authors found that duplication of 1q21.1 was more common in TOF cases than in controls, whereas deletion was not. By contrast, deletion of 1q21.1 was more common in cases of non-TOF CHD than in controls, whereas duplication was not. These findings show that duplications and deletions at chromosome 1q21.1 exhibit a degree of phenotypic specificity in CHD, and they implicate the* GJA5* gene, which is within the approximately 1 Mb critical region, as the gene responsible for the CHD phenotypes that result from copy number imbalances at this locus. Surprisingly, our findings revealed a novel association between a deletion at the 1q21.1 region, including the* GJA5* gene, and TOF in one case (108). Additional phenotypic details and molecular assessments within the critical region of this subject may better define the relationship between the deletion in 1q21 and TOF in this single case.

The 16p11.2 CNV (breakpoint 4-5, BP4-BP5, 29.6–30.2 Mb-Hg19) was identified in case 269, and its phenotypes are characterized by both reciprocal and overlapping deficits that include energy imbalance, language impairment, autism spectrum disorder (ASD), and schizophrenia (SZ). The 16p11.2 deletion includes the region known as the “16p11.2 autism susceptibility locus” (http://omim.org/entry/611913). This deletion includes here approximately 33 genes, from* LOC388242* to* MAPK3*. Three of the 33 genes are recorded in OMIM as disease causing, namely,* KIF22*,* PRRT2,* and* ALDOA*. But the included gene* KCTD13* may also be relevant, as likely candidate for autism spectrum disorder (ASD) phenotype. Malformations or major medical problems can be present in 16p11.2 deletions; however, no specific recurrent malformation sequence or multisystemic involvement is observed [35]. Both 16p11.2 deletion and duplication are associated with ASD, whereas only the duplication is enriched in schizophrenic cohorts. Furthermore, multiple congenital anomalies (MCAs) can be caused by recombination between homologous segmental duplications, leading to microdeletions including 16p11.2.

Ghebranious et al. [36] described a novel* de novo* microdeletion at 16p11.2 in male monozygotic twins who presented with aortic valve abnormalities, seizure disorder, and mild mental retardation. Using array-based comparative genomic hybridization, these authors mapped the microdeletion to the short arm of chromosome 16 at 16p11.2 and refined it using hemizygosity mapping to a region of approximately 0.59 Mb that overlaps with 24 genes. Based on the phenotypes in which the twins presented and based on what is known about the genes within the 16p11.2 microdeletion, the authors identified genes that are likely to be involved in the normal development of the aortic valve, as well as the development of seizure disorders and mental retardation. They postulated that the most probable mechanism for this genetic anomaly is through intrachromosomal recombination between the two 16 homologous, 147 kb segmental duplications. Moreover, they speculate that the HIRA interacting protein 3 (*HIRIP3*) is a candidate gene for aortic stenosis and the seizure related six homolog (mouse)-like 2 isoform (*SEZ6L2*) gene and/or the quinolinate phosphoribosyl transferase (*QPRT*) gene for the seizure disorder. Puvabanditsin et al. [37] used array-CGH to map a deletion of chromosome 16p11.2 that is associated with endocardial fibroelastosis (EFE) in a female infant who died at 3.5 hours of age, and their analysis revealed an 8-oligonucleotide deletion of 0.55 Mb within 16p11.2. The authors reported the first case of congenital primary endocardial fibroelastosis, a rare and poorly understood disease of the endocardium, associated with a previously described microdeletion at 16p11.2. To the best of our knowledge, no duplication of 16p11.2 (29.6–30.2 Mb-Hg19) has yet been associated with isolated TOF. The case identified in our study (269) is an infant, and it is expected that clinical follow-up will yield additional phenotypic details that will better define the relationship between phenotype and the genotype at the 16p11.2 critical region.

The other 3 cases of TOF (56, 137, and 360) showed CNVs at 1p35.1-p34.3, 6q25.2, and 7q31.1, respectively. In the 2 subjects with TGA (49 and 126), we found an interstitial deletion at 7q31.1 and an interstitial deletion at 7q11.21, respectively. These CNVs were classified as variants of unknown significance because they did not affect genes or regions known to be involved in CHD. The clinical relevance of these 5 CNVs among the 10 cases with potentially meaningful genomic imbalances remains uncertain at present, as there is insufficient evidence to determine if the CNVs are related to CHD. Indeed, it can be challenging to determine whether a CNV without a record of clinical importance causes birth defects. We should also consider the possibility that the CNV may have been inherited from a healthy parent; in this case, the CNV could be a pathogenic variant with incomplete penetrance or a benign familiar variation. The highly variable nature of the genome means that care must be taken in assigning pathogenicity to CNVs detected via array-CGH. Parental studies on the CNVs classified as VOUS in this study may enable clinical interpretation and provide valuable information for genetic counseling and the management of disease outcomes. The recurrence risk for many CHDs ranges from 2 to 6%, although this risk increases significantly when the parents are balanced carriers of genomic imbalances [38].

Indeed, it is important to report data on genomic imbalances whose clinical significance is unclear because some of the data may represent recurrent CNVs that could be associated with CHD. Reports of subjects with similar genomic imbalances, as well as clinical findings, may also lead to the identification of newly recognized genomic disorders or candidate genes associated with congenital heart defects [19, 23, 38–42]. However, as the number of recognized CHDs associated with genetic syndromes and chromosomal abnormalities grows and as the clinical characteristics of those syndromes overlap, it will be more difficult to infer precisely which genomic imbalances are associated with heart defects.

This study reports the detection of benign CNVs in genomic regions that consistently harbor common variants. Overall, polymorphic copy number changes were detected in 13 of 60 subjects (21.6%). Of those polymorphisms, we identified seven deletions involving the 5q11.2, 8p11.2, and 10q26.3 chromosome regions and six duplications involving the 7p21.2, 14q32.33, 15q11.2, Xp11.23, and Xq27.2 chromosome regions. Therefore, these variants are unlikely to contribute to the cardiac phenotypes of these individuals.

The detection of common and rare CNVs has generated questions concerning the phenotypic effects of CNVs in CHD and its recurrence risk [43–45]. CNVs represent an important source of genetic variation and have been described as major contributors to phenotypic diversity and disease [46–50]. Recurrent CNVs can occur in both patients and healthy individuals, and frequently, more than one unique CNV is identified in a patient. In the presence of other CNVs or SNPs, a given change in copy number with a high pathogenic penetrance might reduce or aggravate the clinical phenotype. For example, Girirajan and Eichler [51] demonstrated that, as a single event, the 16p11.2 microdeletion predisposes individuals to neuropsychiatric phenotypes, and in association with other large deletions or duplications, it aggravates neurodevelopmental phenotypes.

Currently, the molecular detection of large numbers of CNVs in individuals with CHD as well as healthy individuals is prone to diagnostic pitfalls due to difficulties in interpretation [52]. Most chromosomal abnormalities have clinical effects; however, with the increasing resolution of genomic analysis, the number of instances of benign genomic imbalances has also increased. On a chromosomal or molecular genetic level, CNVs can be expected in every individual [53]. Thus, the identification of variants of unknown clinical significance is expected to increase significantly, particularly as many individuals now have their entire genomes sequenced [54, 55]. Indeed, segmental chromosome regions that might be present in the genome in variable numbers without phenotypic consequences are constantly being identified [56].

Although common strategies have been proposed to help interpret the findings of genomic imbalances associated with CHD [52], there are no universal criteria thus far. Thus, it is essential to have the most accurate and up-to-date information on the clinical significance of known genomic imbalances and CNVs, pathogenic mutations, polymorphisms, and nongenetic factors that may lead to congenital heart defects. However, caution must be taken in interpreting array-CGH results as they relate to CHD. The individuals with conotruncal heart defects who were included in our study were referred to a regional cardiology referral center. Unless a geneticist saw them as outpatients, further consultations at genetics clinics and extended analysis of family members may be necessary to provide accurate clinical examinations, genetic counseling, and calculation of the recurrence risk.

Ours and other studies have shown that an array-CGH approach can be successfully used to detect genomic imbalances in individuals with syndromic and nonsyndromic conotruncal heart defects [19, 23, 25, 38–40, 42, 57, 58]. Overall, array-based genomic investigations have been shown to detect genomic imbalances in between 4 and 27% of syndromic and nonsyndromic CHD cases referred for analysis [23, 38–42, 57, 58]. Our study identified causal or unclassified CNVs in 16.6% (10/60) of cases, which is similar to the range reported in previous studies. Differences in this range between studies may reflect differences in the resolution of the array platforms used, the number of cases included, the criteria used for patient selection, and the interpretation of the relevance of the identified CNVs. Although genome-wide screening for deletions and duplications using array-CGH is a powerful research tool, the frequency of detectable causal CNVs related to conotruncal heart defects depends on the quality of the phenotyping, the local practice, and the availability of funding. For example, in this study, we should consider the subjects' limited access to appropriate clinical genetic diagnosis and care, which is the case in most regions in low- and middle-income countries [59].

Most CNVs are deletions or duplications that arise* de novo* as either unique or recurrent events [24]. One limitation of this study was the inability to distinguish* de novo* and inherited genomic imbalances due to the unavailability of parental DNA.* De novo* CNVs in clinically significant regions of the genome are more likely to be CHD-causative. However, inherited CNVs in known pathogenic regions should not be excluded as causes of congenital heart defects because of the possibility of variable expression and incomplete penetrance within families. Causal CNVs may be inherited from an apparently normal parent and contribute to the abnormal phenotype of the child. These types of CNVs are thought of as susceptibility loci because they increase the chance of a child developing congenital anomalies but may not be sufficient to cause a phenotype themselves. To determine if the CNV findings reported here represent CNVs that arose* de novo* or were inherited, family studies should be recommended for individuals with CHD for whom clinically significant findings were reported.

To date, one critical goal of genetic analysis has been to distinguish benign genomic imbalances from similar-looking causal imbalances associated with CHD. To facilitate the interpretation and analysis of information obtained using cytogenomic approaches, public databases have been developed and are constantly being updated. Nevertheless, many genomic imbalances are novel or extremely rare, making their interpretation problematic and uncertain. Thus, further cytogenomic screening of large CHD patient cohorts with common phenotypic features will contribute to the ongoing development of genotypic-phenotypic correlations, identifying CNVs in dosage-sensitivity genes and defining their locations in the human genome. The technology used to study genomic imbalances has also rapidly expanded [60], and the number of copy number changes and genomic rearrangements in the human genome are likely unlimited. Therefore, comprehensively collecting, organizing, and maintaining raw genotypic and phenotypic data on CHD [18] through various approaches represent a major challenge. To improve the scientific knowledge and medical care related to CHD, national, regional, and international guidelines on data interpretation and clinical management should be implemented to improve expertise and experience in laboratory and clinical praxis.

Of the 60 analyzed subjects, there were 28 (46.6%) whose diagnosis of conotruncal heart defects was associated with other clinical problems, including developmental delays, dysmorphisms, and congenital malformations. Therefore, the other 32 subjects could be described as having isolated conotruncal heart defects, and of those, 5 (15.6%) presented abnormalities that were detected by array-CGH (cases 49, 58, 108, 137, and 269) (Table 2). We believe that the subjects' limited access to appropriate genetic diagnosis and care in our region could have had an influence on this frequency. Thus, we should not exclude the alternate possibility of inaccurate phenotyping in the cases of isolated conotruncal heart defects, as it is possible that other clinical problems, especially those related to minor congenital anomalies, were not identified. Furthermore, one weakness of our retrospective study is the limited clinical information available. We retrieved the clinical information for each subject from the hospital records of their initial referral. Most of the data were recorded at the time of the first cardiological evaluation and were therefore preliminary. Although the clinical presentation of causal abnormalities such as 1q21.1 deletion and 16p11.2 duplication, which were detected in our study, can be extremely variable, we should not exclude the possibility that a subsequent detailed physical examination might detect the presence of subtle phenotypic changes that were not evident at the first examination. CNV detection combined with precision phenotyping may lead to an increased molecular understanding of etiological pathways in these cases.

As is shown by Erdogan et al. [23] and Hightower et al. [42], considering the wide range of observable CHDs, the number of conotruncal heart defects analyzed in our study was relatively small. Moreover, we should consider the selection bias of these studies, as individuals who may have been diagnosed by other means, such as fluorescence* in situ* hybridization or multiplex ligation-dependent probe amplification [27], were not included. Therefore, individuals with deletions in the 22q11.2 locus, which is recognized as being a frequent genetic cause of CHD, were removed from these studies. Nevertheless, we consider the cohort from our study to be representative of individuals with conotruncal heart defects. Our study also demonstrated the feasibility and usefulness of array-CGH analysis to identify copy number changes from subjects with congenital conotruncal heart defects of unknown cause. It was shown that 16.6% (10/60) of subjects possessed causal or unclassified genomic imbalances. Cytogenomic analysis allows professionals to detect genomic imbalances that are consistent with a clinical disorder, and in some cases, this analysis can be performed at an earlier age when only a few clinical findings are clear. Some of the cases presented in our study represent the “diagnostic odyssey” faced by families, with a conclusive diagnosis being reached only after a genomic evaluation is performed. The management of CHD can be greatly affected by an early diagnosis, and it may also permit improvement in genetic counseling for adults affected by CHD who are contemplating reproductive choices.

## 4. Conclusions

In conclusion, although the interpretation of the results must be refined and although there is not yet a consensus regarding which types of CHD would benefit from using cytogenomic analysis as a first-line test, the identification of copy number changes in subjects with conotruncal congenital heart defects can potentially assist in the evaluation and management of this condition. The use of retrospective or prospective array-CGH as a diagnostic tool would benefit families by providing a more accurate diagnosis and would affect overall disease management in a significant number of cases. Furthermore, the results of such studies emphasize the growing importance of the use of genome-wide assays to identify CNVs in subjects with CHD, thereby increasing the number of genomic data sets associated with this condition.

## Figures and Tables

**Table 1:** Study subjects grouped according to the type of congenital heart defect and the male/female distribution (M : F).

Congenital heart defect	*n* (%)	M : F
Tetralogy of Fallot	36 (60)	21 : 15
Transposition of great arteries	22 (36.7)	14 : 8
Truncus arteriosus	2 (33.3)	0 : 2
Total	60 (100)	35 : 25

**Table 2:** Details of the 10 relevant genomic imbalances detected in biorepository samples from subjects with conotruncal heart defects using array-CGH 60.

Case	Gender/age^*∗*^	Del/Dup	Chromosome region	Genomic coordinates (hg 19)	Size (Mb)	Type of CNV	Heart defect	Associated clinical features
268	M/5 y	Del	1p36.33-p36.32	852863–3800088	2.94	Causal	TOF	Prominent forehead, epicanthic folds, flat, broad, and short nose, and downturned corners of the mouth
56	M/11 y	Dup	1p35.1-p34.3	34174663–35055122	0.88	VOUS	TOF	CNS abnormalities
108	M/18 y	Del	1q21.1-q21.2	146641601–147786706	1.15	Causal	TOF	None
137	M/32 y	Dup	6q25.2	153543129–154567984	1.0	VOUS	TOF	None
376	F/13 y	Del	7p22.3-p22.1	707018–5270759	4.56	Causal	TOF	Hypertelorism, epicanthic folds, and micrognathia developmental delay
126	F/5 y	Del	7q11.21	64691936–65070919	0.37	VOUS	TGA	Minor dysmorphic facial features
360	M/2 y	Del	7q31.1	110980176–111202026	0.22	VOUS	TOF	Bilateral inguinal hernias
49	M/14 y	Del	7q31.1	111201968–111304031	0.10	VOUS	TGA	None
58	M/3 y	Del	22q11.21	18919942–21440514	2.5	Causal	TOF	None
269	F/2 y	Dup	16p11.2	29673954–30197341	0.52	Potentially causal	TOF	None

^*∗*^Age at the time of blood collection; array-CGH: microarray-based comparative genomic hybridization; Del: deletion; Dup: duplication; CNS: central nervous system; CNV: copy number variant; M: male; F: female; TOF: Tetralogy of Fallot; TGA: transposition of great arteries; VOUS: variant of uncertain significance.
